# Microglia‐dependent protective and risk signatures driven by Alzheimer's disease genetic factors

**DOI:** 10.1002/alz70862_110127

**Published:** 2025-12-23

**Authors:** Ozkan Is, Jianna Tan, Jeremiah Bergman, Xue Wang, Frederick Q Tutor‐New, Yuhao Min, Zachary Quicksall, Merve Atik, Joseph S. Reddy, Junli Gao, Wei Tsai, Katie D. Sotelo, Thuy T. Nguyen, Takahisa Kanekiyo, Dennis W. Dickson, Mariet Allen, Nilüfer Ertekin‐Taner

**Affiliations:** ^1^ Mayo Clinic, Jacksonville, FL USA; ^2^ Mayo Clinic Graduate School of Biomedical Sciences, Florida, FL USA

## Abstract

**Background:**

Alzheimer's disease (AD) affects all brain cells and has complex genomic and immunological alterations. Previous research discovered missense AD risk or protective variants in microglial genes *ABI3* and *PLCG2*, respectively. Expression levels of these genes are altered in AD and can influence microglial function. This study aims to uncover protective, and risk microglial molecular signatures associated with these variants to determine their role in microglial subtypes and states in AD by single cell expression and functional studies.

**Method:**

We generated microglia‐enriched snRNAseq data from donors harboring either AD protective PLCG2 or AD risk ABI3 missense mutations, or neither mutation. After standard snRNAseq QC, we performed differential expressed gene (DEG) analysis of all microglial cells between variant carriers and non‐carriers using MAST. We defined protective signature as genes that are both down in *ABI3* and up in *PLCG2* mutation‐carriers. In contrast, risk signature genes are up in *ABI3* and down in *PLCG2* mutation‐carriers. We investigated the conservation of protective and risk signatures across multiple datasets, including scRNAseq data from iPSC‐derived microglia cells carrying *PLCG2* protective variant, snRNAseq data from AD‐resilient donors, and data from AD and other diagnostic groups across multiple brain regions sourced from external datasets.

**Result:**

We obtained snRNAseq profiles of 35,000 microglia from AD variant carriers. Our DEG analysis among all microglia cells provided 227 microglial protective and 293 risk signature genes defined by these variants. Using integrated analysis of multiple internal and external datasets, we further narrowed down these signatures. We determined that these high‐confidence protective signature genes are downregulated in early AD, upregulated in late AD brains and positively‐correlated with protective variant load in in vitro models. In contrast, risk signature genes are upregulated in early AD, downregulated in late AD and resilient donors. Risk signature expression is decreased with protective PLCG2 variant load, and altered with Aβ treatment in in vitro models.

**Conclusion:**

Our study uncovers microglia specific protective and risk signatures associated with AD using sn/scRNAseq datasets from multiple sources and models. These findings nominate novel immune targets and pathways with implications for microglial function in health and disease, and ultimately therapeutic potential.

